# Antibiotic Resistance Patterns of *Pseudomonas* spp. Isolated From Raw Milk Revealed by Whole Genome Sequencing

**DOI:** 10.3389/fmicb.2020.01005

**Published:** 2020-06-03

**Authors:** Lu Meng, Huimin Liu, Tu Lan, Lei Dong, Haiyan Hu, Shengguo Zhao, Yangdong Zhang, Nan Zheng, Jiaqi Wang

**Affiliations:** ^1^Laboratory of Quality and Safety Risk Assessment for Dairy Products of Ministry of Agriculture and Rural Affairs, Institute of Animal Sciences, Chinese Academy of Agricultural Sciences, Beijing, China; ^2^Key Laboratory of Quality & Safety Control for Milk and Dairy Products of Ministry of Agriculture and Rural Affairs, Institute of Animal Sciences, Chinese Academy of Agricultural Sciences, Beijing, China; ^3^College of Animal Science and Technology, Anhui Agricultural University, Hefei, China

**Keywords:** *Pseudomonas* spp., antibiotic resistance, whole genome sequencing, milk, multiple antibiotic resistance index

## Abstract

Psychrotrophic bacteria in raw milk are most well known for their spoilage potential and the economic losses they cause to the dairy industry. Food-related psychrotrophic bacteria are increasingly reported to have antibiotic resistance features. The aim of this study was to evaluate the resistance patterns of *Pseudomonas* spp. isolated from bulk-tank milk. In total, we investigated the antibiotic susceptibility profiles of 86 *Pseudomonas* spp. isolates from raw milk. All strains were tested against 15 antimicrobial agents. *Pseudomonas* isolates were most highly resistant to imipenem (95.3%), followed by trimethoprim-sulfamethoxazole (69.8%), aztreonam (60.5%), chloramphenicol (45.3%), and meropenem (27.9%). Their multiple antibiotic resistance (MAR) index values ranged from 0.0 to 0.8. Whole-genome sequencing revealed the presence of intrinsic resistance determinants, such as BcI, *amp*C-09, *bla*CTX-M, *opr*D, *sul*1, *dfr*E, *cat*A1, *cat*B3, *cat*I, *flo*R, and *cml*V. Moreover, resistance-nodulation-cell division (RND) and ATP-binding cassette (ABC) antibiotic efflux pumps were also found. This study provides further knowledge of the antibiotic resistance patterns of *Pseudomonas* spp. in milk, which may advance our understanding of resistance in *Pseudomonas* and suggests that antibiotic resistance of *Pseudomonas* spp. in raw milk should be a concern.

## Introduction

*Pseudomonas* spp., which have been identified as predominantly psychrotrophic bacteria, are important spoilage bacteria in food (Marchand et al., 2009). The *Pseudomonas* genus is found extensively in environments such as water, soil, and sediment (Devarajan et al., 2016). They are ubiquitous Gram-negative bacteria that have a wide metabolic versatility, which allows them to acclimate to different habitats with temperatures ranging from 4 to 42°C (Quigley et al., 2013; Molina et al., 2014; Chakravarty and Gregory, 2015). Moreover, *Pseudomonas* can multiply at cold temperatures and accounts for more than half of all bacteria found in milk (Munsch-Alatossava and Alatossava, 2006; Fricker et al., 2011; Von Neubeck et al., 2015). *Pseudomonas* strains produce heat-stable extracellular peptidases and/or lipases that spoil raw milk, and some strains are opportunistic pathogens that are found in the environment, such as *Pseudomonas aeruginosa* (Jeukens et al., 2017).

The widespread administration of antimicrobial agents to animals used for food products has imposed a strong selective pressure that increases resistance among known pathogens and commensal bacteria (Beena et al., 2011; Decimo et al., 2016). Antimicrobial resistant *Pseudomonas* is also a concern as antimicrobial resistance is a key factor in the emergence of infectious diseases (Jones et al., 2008; Gomi et al., 2017). A study based on phenotypes and 16S rDNA gene sequences identified *Pseudomonas* isolates that were categorized as being a high risk for antibiotic resistance (Munsch-Alatossava et al., 2012). Moreover, *Pseudomonas* spp. have the ability to remain viable in the aquatic environment for long periods owing to innate resistance mechanisms, thereby increasing the risk of spreading antibiotic resistance genes and mobile genetic elements (Devarajan et al., 2016). The exchange of genetic material encoding resistance genes via mobile genetic elements, plasmids, or transposons can result in transfer of resistance between pathogenic and non-pathogenic bacteria (Mathur and Singh, 2005; Panelists, 2006; Decimo et al., 2016). Yomoda et al. (2003) reported that 22 of 32 *Pseudomonas putida* strains isolated from Japanese hospitals carried plasmids that could be transferred to *P. aeruginosa* by conjugation or transformation. Therefore, the transfer of antibiotic resistance determinants from non-pathogenic species to pathogens is a serious concern (Yomoda et al., 2003; Molina et al., 2014).

Antibiotic resistance has been a focus of research on food-related and mastitis pathogens in milk for many years. The potential for non-pathogenic commensal foodborne bacteria to become a biological hazard has also been investigated as resistance genes can be spread to humans via food (Decimo et al., 2016). The aim of this study was to evaluate the resistance of *Pseudomonas* spp. isolated from bulk-tank milk in China to different antibiotics. This study represents an extensive investigation of resistance of *Pseudomonas* spp. to a number of human and veterinary antimicrobial agents.

## Materials and Methods

### Bacterial Isolates

A total of 143 *Pseudomonas* strains were isolated from raw milk samples from 87 bulk tanks in Shaanxi province, China, in spring 2016 (average daily temperature >20°C) for a previous study of their proteolytic properties (Meng et al., 2017). All isolates were re-identified using the *rpo*D gene as *rpo*D provides higher resolution than 16S rDNA gene sequences for *Pseudomonas* species (Rajwar and Sahgal, 2016). In total, 86 isolates representing 11 different *Pseudomonas* spp. were subjected to antimicrobial susceptibility testing. A brief description of these 86 isolates is provided in Supplementary Table S1.

### Antimicrobial Susceptibility Testing

Antimicrobial susceptibility tests were performed using broth microdilution with cation-adjusted Mueller–Hinton broth (BD, Franklin Lake, NJ, United States) following Clinical and Laboratory Standards Institute (Clinical and Laboratory Standards Institute [CLSI]) guidelines (2018). The antimicrobials used for susceptibility testing included 15 antimicrobial agents belonging to the following classes: penicillins (ampicillin, penicillin), monobactams (aztreonam), cephamycins (cefoxitin), phenicols (chloramphenicol), fluoroquinolones (ciprofloxacin, levofloxacin), lincosamides (clindamycin), lipopeptides (polymyxin B), aminoglycosides (gentamicin), carbapenems (imipenem, meropenem), tetracyclines (tetracycline), folate pathway inhibitors (trimethoprim-sulfamethoxazole), and β-lactams (ceftiofur). Of these antibiotics, ampicillin, penicillin, cefoxitin, ciprofloxacin, gentamicin, tetracycline, and ceftiofur are used as veterinary medicines (MOA, 2002). Our results were interpreted according to European Committee on Antimicrobial Susceptibility Testing (European Committee on Antimicrobial Susceptibility Testing [EUCAST], 2018) and Clinical and Laboratory Standards Institute [CLSI] criteria (2016, 2018). Multidrug-resistant (MDR) strains were defined as being resistant to three or more antimicrobial classes. However, because neither European Committee on Antimicrobial Susceptibility Testing [EUCAST] (2018) nor Clinical and Laboratory Standards Institute [CLSI] (2018) criteria provide minimal inhibitory concentration (MIC) breakpoints for ampicillin, penicillin, cefoxitin, clindamycin, or ceftiofur for non-*Enterobacteriaceae*, results for these five antibiotics are provided as MIC values.

The multiple antibiotic resistance (MAR) index for each isolate was determined for 10 antimicrobial agents according to Blasco et al. (2008):

$$\begin{array}{rcl} & & \mathrm{MAR}\;\mathrm{index}\\ & & \;=\frac{\mathrm{Number}\;\mathrm{of}\;\mathrm{antibiotics}\;\mathrm{to}\;\mathrm{which}\;\mathrm{isolate}\;\mathrm{was}\;\mathrm{resistant}}{\mathrm{Total}\;\mathrm{number}\;\mathrm{of}\;\mathrm{antibiotics}\;\mathrm{tested}} \end{array}$$

### Whole Genome Sequencing

Strains exhibiting the same antimicrobial resistance patterns were excluded from sequencing analysis. After exclusion, 44 isolates were selected for sequencing. The genomic DNA of each isolate was extracted using a Wizard^®^ Genomic DNA Purification Kit (Promega Corporation, Fitchburg, WI, United States) following the manufacturer’s protocol. DNA samples were sheared into 400- to 500-bp fragments using a Covaris M220 Focused-Ultrasonicator (ThermoFisher Scientific, Waltham, MA, United States) according to the manufacturer’s protocol. Illumina sequencing libraries were then prepared from the sheared fragments using a NEXTflex^TM^ Rapid DNA-Seq Kit (Bioo Scientific, Austin, TX, United States). Briefly, 5’ prime ends were first end repaired and phosphorylated. Next, 3’ ends were A-tailed and ligated to sequencing adapters. Adapter-ligated products were then enriched using PCR. Finally, the prepared libraries were used for paired-end Illumina sequencing (2 × 150 bp) on an Illumina HiSeq X Ten machine (Illumina, San Diego, CA, United States).

### Analysis of Sequence Data

Data generated from the Illumina platform were used for bioinformatics analysis. All analyses were performed using the I-Sanger Cloud Platform^1^ from Shanghai Majorbio BioTech Co., Ltd. (Shanghai, China). Briefly, the original image data were transferred into sequence data, defined as raw data or raw reads, by base calling and saved as a FASTQ file. A quality statistic was then applied for quality trimming to remove low-quality data from the clean data. Assembly of the clean reads was performed using SOAPdenovo2 (Luo et al., 2015). Finally, resistance genes were detected using the ResFinder web server and ARG-ANNOT database (Zankari et al., 2012; Gupta et al., 2014). All whole genome sequence data from this study have been deposited in the NCBI Sequence Read Archive database (accession numbers PRJNA523883, PRJNA523885, and PRJNA593738).

## Results

### Antimicrobial Susceptibility Profiles of *Pseudomonas* spp.

**FIGURE 1:**
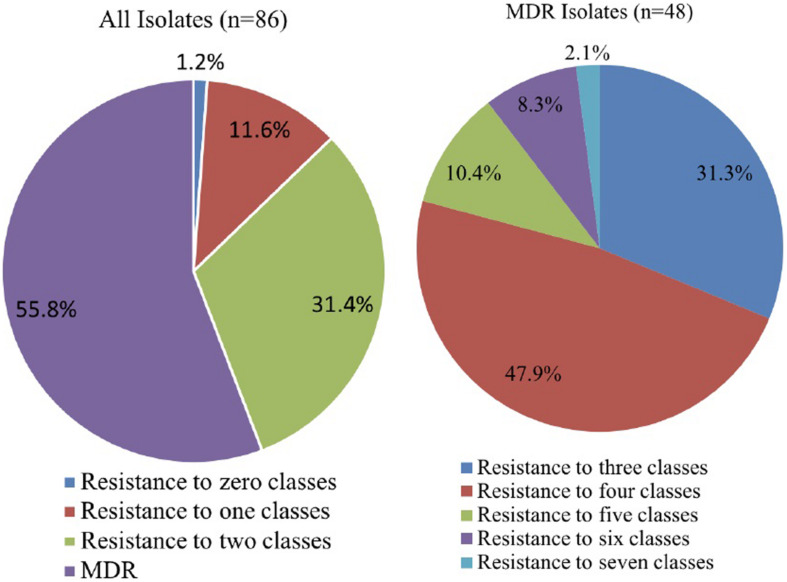
Distribution of resistance patterns.

Isolates were tested for susceptibility to 15 antibiotics. Results were first interpreted according to European Committee on Antimicrobial Susceptibility Testing [EUCAST] (2018), followed by Clinical and Laboratory Standards Institute [CLSI] (2016, 2018). Table 1 shows the *Pseudomonas* spp. that were found to belong to different interpretive categories, including susceptible (S), intermediate (I), and resistant (R), for which MIC breakpoints are available). Wide variability was observed in the susceptibility of isolates to gentamicin, ciprofloxacin, tetracycline, and levofloxacin. Approximately 95% of *Pseudomonas* strains were susceptible to gentamicin and ciprofloxacin, followed by 93.0% that were susceptible to tetracycline and 91.9% that were susceptible to levofloxacin. However, a high percentage of *Pseudomonas* strains were resistant to imipenem (95.3%). Resistance to trimethoprim-sulfamethoxazole (69.8%), aztreonam (60.5%), chloramphenicol (45.3%), and meropenem (27.9%) was also widespread in this study. The distribution of resistance patterns to the 10 antibiotics tested is shown in Figure 1. In total, 48 (55.8%) isolates were resistant to three or more drug classes and were defined as MDR strains. One isolate was not resistant to all eight classes of antibiotics, and one isolate could resist seven classes of antibiotics. Moreover, the 86 tested isolates had MAR index values ranging from 0.0 to 0.8 (Supplementary Table S2).

**TABLE 1:** The interpretive categories of *Pseudomonas* strains to the tested antibiotics.

Class	Antibiotic	References	Interpretive	Total number (*n* = 86)
Monobactam	aztreonam	European Committee on Antimicrobial Susceptibility Testing [EUCAST], 2018	S	–
			I	34
			R	52
Phenicols	chloramphenicol	Clinical and Laboratory Standards Institute [CLSI] (2018)	S	31
			I	16
			R	39
Fluoroquinolones	ciprofloxacin	European Committee on Antimicrobial Susceptibility Testing [EUCAST], 2018	S	81
			I	–
			R	5
	levofloxacin	European Committee on Antimicrobial Susceptibility Testing [EUCAST], 2018	S	79
			I	–
			R	7
Aminoglycosides	gentamicin	European Committee on Antimicrobial Susceptibility Testing [EUCAST], 2018	S	82
			I	–
			R	4
Carbapenems	imipenem	European Committee on Antimicrobial Susceptibility Testing [EUCAST], 2018	S	1
			I	3
			R	82
	meropenem	European Committee on Antimicrobial Susceptibility Testing [EUCAST], 2018	S	41
			I	21
			R	24
Tetracyclines	tetracycline	Clinical and Laboratory Standards Institute [CLSI] (2018)	S	80
			I	2
			R	4
Folate Pathway Antagonists	trimethoprim-sulfamethoxazole	Clinical and Laboratory Standards Institute [CLSI] (2018)	S	26
			I	–
			R	60
Lipopeptides	polymyxin B	Clinical and Laboratory Standards Institute [CLSI] (2016)	S	72
			I	5
			R	9
*No MIC Breakpoints*				
Penicillins	ampicillin	–	<256	35
			⩾256	51
	penicillin	–	<256	2
			⩾256	84
Cephems	cefoxitin	–	<256	1
			⩾256	85
β-lactam	ceftiofur	–	⩽32	39
			64	25
			⩾128	22
Lincosamides	clindamycin	–	<256	3
			⩾256	83

*S is susceptible, I is intermediately resistant, and R is resistant.*

For the antibiotics ampicillin, cefoxitin, ceftiofur, clindamycin, and penicillin, no European Committee on Antimicrobial Susceptibility Testing [EUCAST] (2018) or Clinical and Laboratory Standards Institute [CLSI] (2016, 2018) breakpoints are available for *Pseudomonas* spp. We found MIC values ≥256 μg/mL for 85 isolates for cefoxitin, 84 isolates for penicillin, and 83 isolates for clindamycin. All MIC values are shown in Supplementary Table S1.

### Resistance Determinants

Overall, more than 100 resistance determinants were found in 44 isolates (Table 2), and all of the isolates carried resistance determinants. Eleven different aminoglycoside resistance genes were detected. The most prevalent aminoglycoside resistance gene, *aph(6)-Ic*, is one of three resistance genes present in the composite transposon Tn*5*, which is found in Gram-negative bacteria (Steiniger-White et al., 2004). The *ksg*A gene, which confers resistance to kasugamycin (Zou et al., 2018), was also found in seven isolates. Genes *ant(3”)-Ia, aph(6)-Id* and *aph(3’)-IIc* as well as *rps*L mutations, known to be responsible for streptomycin resistance (Ramirez and Tolmasky, 2010), were also found in these isolates (Supplementary Table S3).

**TABLE 2:** Resistance determinants detected in 44 isolates.

Antibiotic class	Resistance determinant (no. of isolates)
Aminocoumarin	*alaS* (2), *cysB* (2), *gyr*B (3), *nov*A (3), *mdt*A (2), *mdt*B (2), *mdtC* (8)
Aminoglycoside	*aac(6’)*-*IIa* (1), *aad*A11 (1), *aad*A (1), *aph(3”)*-*Ib* (2), *aph*(3*’*)-*IIc* (2), *aph(3’)-Ib* (2), *aph*(6)-*Ic* (7), *aph*(6)-*Id* (2), *ksg*A (6), *rps*L (2), SAT-3 (2)
β-Lactam	BcI (1), *amp*C-09 (4), *bla*_CTX–M_ (1), *bla*_CMY–__51_ (2), LRA-2 (2), LRA-13 (4), *mec*C (1), OCH-8 (5), PBP1a (2), PBP1b (1), PBP2 (1), PDC-9 (2), SFH-1 (1), *opr*D (2)
Fluoroquinolones	*gyr*A (3), *gyr*B (1), *pat*A (2), *pat*B (2), *par*C (2), *parE* (2), *qnr*B (5), *emr*A (1), *emr*B (2), *mdt*K (1), *mfd* (2)
Fosfomycin	*mdt*D (2), *mdt*G (6), *mdt*H (1), *glp*T (2), *mur*A (2)
Glycopeptides	*van*A (2), *van*F (3), *van*G (3), *van*HA (2), *van*HB (2), *van*HD (2), *van*HF (2), *van*HO (2), *van*L (1), *van*M (2), *van*O (1), *van*RB (2), *van*RF (2), *van*RI (2), *van*SA (1), *van*SB (2), *van*SE (2), *van*SI (1), *van*SM (2), *van*SN (2), *van*SO (2), *van*TG (2), *van*TrL (2),
Lincosamide	*Erm(47)* (2), *lmr*C (3), *lmr*D (1)
Lipopeptides	*arn*A (10), *bac*A (35), *bcr*A (2), *lia*R (2), *lia*S (2), *mpr*F (3), *pho*P (11), *pho*Q (11), *pmr*A (2), *pmr*B (2), *pmr*C (2), *pmr*E (2), *pmr*F (10), *ros*A (8), *ros*B (8), *flo*R (2), *lpx*A (2), *lpx*C (2), *cls* (2), *pgs*A (2), *rpo*C (2)
Macrolide	*car*A (2), *mac*A (10), macB (11)
Macrolide-Lincosamide- Streptogramin B	*mef*A (1)
Phenicol	*cat*A1 (1), *cat*B3 (1), *cat*I (1), *flo*R (1), *cml*V (2)
Rifampin	*rpo*B (11)
Streptogramin	*vat*E (2)
Sulfonamide	*sul*1 (4)
Tetracycline	*otrA* (2), *tet*42 (3), *tet*A (1), *tet*B(P) (2), *tet*G (3), *tet*M (2), *tet*T (2)
Trimethoprim	*dfr*E (1)
Isoniazid	*kat*G (1), *kas*A (2)
Benzenoids	*tri*A (11), *tri*B (11), *tri*C (3), *opm*H (11)

Fourteen different β-lactam resistance genes were detected. Although the total number of β-lactam resistance genes was high, the prevalence of each gene was low. Of the β-lactam resistance determinants, OCH-8 confers resistance to third-generation cephalosporins (Alonso et al., 2017), whereas LRA-13 confers resistance to amoxicillin, ampicillin, cephalexin, and carbenicillin (Silveira et al., 2018). Penicillin-binding proteins (PBPs) are the physiological targets of β-lactam antibiotics (Spratt and Cromie, 1988), and mutations in the PBPs PBP1a, PBP1b, and PBP2, which were found to confer resistance to β-lactams (Di Guilmi et al., 2003; Stanhope et al., 2008; Unemo et al., 2012), have also been detected in four isolates (Table 2 and Supplementary Table S3). In addition, the remaining nine β-lactam resistance genes have been demonstrated to confer resistance to cephalosporin, carbapenem, monobactam, and cephamycin (Carfi et al., 1995; Saavedra et al., 2003; Rodriguez-Martinez et al., 2009; Milheirico et al., 2017).

Another important resistance gene group was found to confer resistance to lipopeptides. In total, 21 resistance genes have been detected with 12 genes conferring resistance to polymyxin, such as *pho*P and *pho*Q. The most common gene, *bac*A, is a bacitracin resistance gene (Shaaly et al., 2013), and *bcr*A is another one, but it is only found in two isolates. *flo*R is a resistance gene against florfenicol (Arcangioli et al., 1999). Additionally, mutations in *liaR*, *lia*S, *cls*, *pgs*A, and *rpo*C, known to be responsible for daptomycin resistance (Yang et al., 2010; Arias et al., 2011; Peleg et al., 2012; Miller et al., 2013), were found in a few strains (Table 2 and Supplementary Table S3).

In total, 16 resistance genes were speculated from other bacteria, such as *gyr*A, *gyr*B, *rps*L, PBP1a, PBP1b, PBP2, *lia*R, *lia*S, *cls*, *pgs*A, *rpo*C, *rpo*B, *kat*G, and *kas*A. These genes confer resistance to aminocoumarin, aminoglycoside, β-lactam, fluoroquinolones, fosfomycin, lipopeptides, rifampin, and isoniazid. Moreover, it is speculated that all the rifampin and isoniazid resistance determinants were transferred from *Escherichia coli*, *Mycobacterium tuberculosis*, and *Staphylococcus aureus.*

In addition to these resistance genes, genes for resistance-nodulation-cell division (RND) and ATP-binding cassette (ABC) antibiotic efflux pumps, including AcrAB-TolC, MexAB-OprM, MexCD-OprJ, MexEF-OprN, MexJK-OpmH, MexMN-OprM, MexPQ-OpmE, MexVW-OprM, and EfrAB, were also detected (Depardieu et al., 2007; Chen and Duan, 2016). These RND efflux pumps can be mediated by the local repressor gene mutations, global regulatory gene mutations, or other mutations except MexAB-OprM and MexXY-OprM, which contribute to intrinsic multidrug resistance in *P. aeruginosa* PAO1 (Morita et al., 2001a; Piddock, 2006).

## Discussion

Bacterial antibiotic resistance is considered a worldwide problem in the medical, environmental, and agricultural fields. Many researchers have focused on antibiotic resistance in pathogenic bacteria, which pose immediate risks to human health. However, more and more interests are focusing on commensal bacteria associated with food (Munsch-Alatossava et al., 2012; Decimo et al., 2016). *Pseudomonas* spp. isolated from raw milk shows extensive resistance to many antibiotics, and some even exhibit the highest known levels of antibiotic resistance (Straley et al., 2006; Munsch-Alatossava and Alatossava, 2007; Decimo et al., 2016).

EUCAST and CLSI criteria are the standards for bacterial resistance assessment. However, clinical breakpoints are based on parameters that are only relevant for therapeutic purposes. Therefore, assessing the antibiotic susceptibility of environmental bacteria using only CLSI criteria is inadequate. In contrast, EUCAST criteria are clinically and/or epidemiologically based and are more reliable for the interpretation of the antibiotic susceptibility of environmental bacteria (Berendonk et al., 2015; Gomi et al., 2017). Therefore, European Committee on Antimicrobial Susceptibility Testing [EUCAST] (2018) criteria were used in the present study to assess the resistance phenotypes of *Pseudomonas* spp. to aztreonam, ciprofloxacin, levofloxacin, gentamicin, imipenem, and meropenem. However, Clinical and Laboratory Standards Institute [CLSI] (2016, 2018) criteria were used to interpret chloramphenicol, polymyxin B, tetracycline, and trimethoprim-sulfamethoxazole resistance as there is no MIC value for these antibiotics in the European Committee on Antimicrobial Susceptibility Testing [EUCAST] (2018) criteria.

According to a previous survey, β-lactam antibiotics are commonly used for dairy mastitis therapy (Liu et al., 2017). Therefore, seven β-lactam antibiotics (ampicillin, aztreonam, cefoxitin, ceftiofur, imipenem, meropenem, and penicillin) were tested in the present study. Around 60% of *Pseudomonas* strains were resistant to aztreonam, which is higher than described by Decimo et al. (2016). A study conducted between 2010 and 2012 reported that 51–100% of *Pseudomonas* spp. in non-turbid drinking water were resistant to aztreonam (Flores Ribeiro et al., 2014). In contrast to our results, which showed that 95.3% of *Pseudomonas* strains were resistant to imipenem, Decimo et al. (2016) found that only 11% of *Pseudomonas* spp. from bulk-tank milk were resistant to imipenem, and Arslan et al. (2011) found that all *Pseudomonas* spp. isolates from cheese were susceptible to imipenem. In the present study, the meropenem resistance rate was only 27.9% (*n* = 24), which is similar to resistance rates reported in previous studies of *Pseudomonas* spp. from bulk-tank milk and the Danube River (Decimo et al., 2016; Kittinger et al., 2016). Although no breakpoints were available for the four other β-lactam antibiotics, we found that 59.3, 98.8, 2.3, and 97.7% of isolates had MIC value ≥256 μg/mL for ampicillin, cefoxitin, ceftiofur, and penicillin, respectively. In the 1980s, studies reported that *P. aeruginosa* that were the dominant cause of nosocomial infections were resistant to almost all aminoglycosides and β-lactams with the exception of cephalosporins and carbapenems (Neu, 1983; Acar, 1985). Penicillin G is still the most commonly used antibiotic for dry cow therapy (United States Department of Agriculture, Animal Plant Health Inspection Service National Animal Health Monitoring System [USDA APHIS], 2008), and one study found that 100% of *Pseudomona*s spp. isolates from cheese were resistant to penicillin G (Arslan et al., 2011).

In the present study, 14 different β-lactamase genes were detected in approximately 20 of the *Pseudomona*s isolates, which were resistant to at least one β-lactams antibiotic from among aztreonam, imipenem, and meropenem except 149-2 and 152-2. Gram-negative bacteria produce AmpC-type β-lactamases that can hydrolyze amino- and ureido-penicillins, cephamycin, and—at low levels—cephalosporin and monobactams (Rodriguez-Martinez et al., 2009). Four isolates carrying *amp*C-09 were all resistant to aztreonam, MIC value for cefoxitin ≥256 μg/mL, and MIC value for cefoxitin ≥64 μg/mL except 198-5. Two isolates 141-6 and 151-5 harbored *bla*_CMY–__51_, an *amp*C variant first described in *Citrobacter freundii* (Porres-Osante et al., 2014), and were resistant to aztreonam, MIC value for cefoxitin ≥256 μg/mL, and MIC value for cefoxitin ≥128 μg/mL. The isolate 114-2 was intermediately resistant to aztreonam with the gene *bla*_CTX–M_, which has been demonstrated to hydrolyze aztreonam (Wang et al., 2011). All five isolates with OCH-8 and LRA-2 showed MIC value 128 μg/mL for ceftiofur. However, five isolates taking BcI, *bla*_CTX–M_, and LRA-13 showed a ceftiofur MIC value for ceftiofur ≤64 μg/mL. The resistance genes that have been found were much lower than the β-lactam resistance rates. Meanwhile, *Pseudomonas* spp. are reported to be naturally resistant to penicillin G as well as the majority of related β-lactam antibiotics (Decimo et al., 2016). The primary mechanisms of protection of *Pseudomona*s against β-lactams are the production of β-lactamases, the decrease or loss of the OprD porin in the outer membrane, and the overproduction of RND efflux pumps (Wolter and Lister, 2013). However, RND efflux pumps contribute to increased resistance to β-lactams, β-lactamase inhibitors, and certain carbapenems, including penicillin, aztreonam, meropenem, and ampicillin but not imipenem, when mediated by the local repressor gene mutations or global regulatory gene mutations. For example, a global regulatory gene, *sox*R, in *P. aeruginosa* has also been described to regulate efflux pumps (Palma et al., 2005; Piddock, 2006). And mutations in the local repressor genes *acr*R, *nfx*B, *mex*S, and *mex*L can induce overexpression of AcrAB-TolC, MexCD-OprJ, MexEF-OprN, and MexJK, respectively (Poole et al., 1996; Chuanchuen et al., 2002; Sobel et al., 2005).

Although no *bla*IMP, *bla*VIM, and *bla*NDM have been detected, carbapenem resistance genes PCD-9 and SFH-1 were found in three isolates. Of these, 68-1 and 103-1 showed resistance to imipenem and meropenem and 106-3 only to imipenem. Moreover, imipenem and meropenem resistance in *P. aeruginosa* have been found to be associated with the loss of the OprD porin combined with the activity of chromosomal β-lactamase (AmpC) (Livermore, 2001; El Amin et al., 2005). However, unlike imipenem, meropenem can be expelled by MexB-mediated efflux (Livermore, 2001). All imipenem-/meropenem-resistant isolates in the present study had *opr*D-loss mutations with the exception of two isolates, 141-6 and 151-5, that were resistant to both imipenem and meropenem but with *opr*D. Fang et al. (2014) demonstrated that reduced expression of *opr*D without *opr*D loss was the predominant mechanism of imipenem resistance.

In addition to β-lactam antibiotics, ciprofloxacin, sulfamethoxazole-trimethoprim, and gentamicin are also frequently used for dairy mastitis therapy in China (Liu et al., 2017). However, *Pseudomonas* spp. in the present study exhibited high susceptibility to fluoroquinolone (ciprofloxacin, 94.2%; levofloxacin, 91.9%) and aminoglycoside (gentamicin, 95.3%) antibiotics (Table 1). Our results confirmed the findings of Munsch-Alatossava and Alatossava (2007) and Decimo et al. (2016), who reported antibiotic resistance patterns of Gram-negative psychrotrophic bacteria from bulk-tank milk in Finland and Italy. However, psychrotrophic isolates from raw and pasteurized milk have been found to be resistant to gentamicin (Beena et al., 2011; Munsch-Alatossava et al., 2012). In addition to the 12 resistance genes found in 86 isolates, the enzymes ANT(3’), APH(3’), APH(6), and RpsL mutations confer resistance to streptomycin (Sreevatsan et al., 1996; Ramirez and Tolmasky, 2010). The gene *aac(6’)-IIa*, which can produce enzymes that actively mediate acetylation of gentamicin, was found in 114-2 that exhibited gentamicin resistance. Moreover, the RND efflux pump MexXY can be induced by sub-inhibitory concentrations of gentamicin and acquire resistance against aminoglycosides when overexpressed (Morita et al., 2001b).

Fluoroquinolone resistance has been associated with mutations in the genes *gyr*A and *par*C (Aoike et al., 2013). The mutations were found in two isolates, 141-6 and 151-5, that were resistant to ciprofloxacin and levofloxacin. Moreover, these two isolates also carried eight other quinolones resistance determinants, including mutations in the quinolone resistance-determining regions of DNA gyrase (*gyr*B) and DNA topoisomerase IV (*par*E). Isolate 103-1, which carried a *gyr*A mutation from *E. coli*, also exhibited resistance to ciprofloxacin and levofloxacin. Five isolates harbored the plasmid-mediated quinolone resistance gene *qnr*B; however, these isolates were all susceptible to ciprofloxacin and levofloxacin. Moreover, 60 isolates were resistant to trimethoprim-sulfamethoxazole. However, only five isolates carried the trimethoprim resistance gene *dfr*E or the sulfamethoxazole resistance gene *sul*1 (Toleman et al., 2007; Chen et al., 2019). Resistance to ciprofloxacin and levofloxacin in the other isolates may have been due to RND efflux pumps (Kohler et al., 1996; Li et al., 2015).

Polymyxin lipopeptide antibiotics are currently last-resort antibiotics for the treatment of MDR Gram-negative bacterial infections. Of 21 lipopeptide resistance genes, 12 confer resistance to polymyxin, including *arn*A, *pho*P, *pho*Q, *pmr*A, *pmr*B, *pmr*C, *pmr*E, *pmr*F, *ros*A, *ros*B, *lpx*A, and *lpx*C, with *pho*P and *pho*Q being the most common (McPhee et al., 2003; Gatzeva-Topalova et al., 2005; Lee and Ko, 2014; Zhang et al., 2017). Polymyxin B resistance has been found to be conferred by mutations in two-component regulatory systems of *pho*PQ, which are well known to contribute to polymyxin resistance in *P. aeruginosa* (Barrow and Kwon, 2009; Miller et al., 2011; Lee and Ko, 2014). The PmrF operon also plays an essential role in resistance to polymyxin and can be upregulated by PhoPQ (Marceau et al., 2004). In the present study, the number of mutations in *pho*PQ regions was higher than polymyxin B resistance rates, and a few polymyxin B–resistant isolates carried the fosmidomycin resistance genes *ros*A and *ros*B, which encode an efflux pump that confers resistance to polymyxin B in response to polymyxin B (Bengoechea and Skurnik, 2000).

In our study, resistance rates of *Pseudomonas* isolates to chloramphenicol and tetracycline were 45.3% (39/86) and 4.7% (4/86), respectively, which was much lower than the ratios of *Pseudomonas aeruginosa* isolated from milk samples and from major hospitals and laboratories in Jamaica (Brown and Izundu, 2004; Decimo et al., 2016). The chloramphenicol resistance is mainly caused by enzymatic inactivation by chloramphenicol acetyltransferases (CAT) and also efflux mechanisms mediated by *cml* genes (Schwarz et al., 2004). In total, *cat* and *cml* genes were found in five isolates, which is less than the number of chloramphenicol resistance isolates (*n* = 39). However, the effects of simultaneous expression of multidrug transporters, such as AcrAB–TolC or MexAB–OprM, can increase the chloramphenicol resistance of *P. aeruginosa* (Lee et al., 2000). Seven different tetracycline resistance genes have been found in this study, of which *otr*A, *tetB*(P), *tet*M, and *tet*T could code ribosomal protection proteins and *tet*42, *tet*A, and *tet*G are tetracycline efflux pump genes (Thaker et al., 2010). However, only two tetracycline-resistant isolates harbored the *tet*G gene. The other two resistant isolates harbored RND efflux pumps, such as MexCD-OprJ, which could help the isolates resistant to tetracycline (Li et al., 2015). The isolates that carried the resistance determinants were not resistant to tetracycline, especially 114-6 and 115-5, which had five and six different resistance genes, respectively. Moreover, MIC values of 83 isolates were ≥256 μg/mL for clindamycin. Gram-negative aerobic organisms are reported to be uniformly resistant to clindamycin, whereas only a few resistance genes have been detected (Ras et al., 1992).

One interesting finding was the presence of a triclosan efflux pump. The genes *tri*A, *tri*B, and *tri*C, which were present in three isolates, are tightly coupled transcripts that form the TriABC protein and associate with OpmH to assemble a functional triclosan efflux pump (Mima et al., 2007). Triclosan is now used in many consumer products, including toothpastes, soaps, cosmetics, cutting boards, and mattress pads (Bhargava and Leonard, 1996; Chuanchuen et al., 2002). Triclosan contamination is raising concerns due to the high risk of it converting into toxic dioxin in aquatic environments, and attention should be paid to its usage (Yan et al., 2019).

In total, 48 isolates were MDR strains with 22 different combined groups (Supplementary Table S1). Although the isolates harbor ARGs, RND efflux pumps may play a role in resistance because they have wide antibiotic substrate spectrums (Depardieu et al., 2007). The MAR index is considered a good risk assessment tool. A MAR index value of 0.20 used to differentiate between low and high risk of contamination (Krumperman, 1983; Riaz et al., 2011). The MAR index values of the 86 isolates tested in this study were between 0.0 and 0.8 with 59.3% (51/86) of isolates having MAR indices >0.20, suggesting high antibiotic use and high selective pressure. Swetha et al. (2017) found that 19 *P. aeruginosa* isolates from raw milk samples had MAR index values ranging from 0.33 to 1.0 and were resistant to between four and 12 antibiotics. *Pseudomonas* isolates from wastewater treatment facilities in South Africa had MAR index values ranging from 0.26 to 0.58 and were resistant to between five and 11 antibiotics (Odjadjare et al., 2012). Moreover, some *Pseudomonas* isolates acquire resistance to antibiotics through horizontal gene transfer (HGT) of plasmids carrying genetic material encoding for antibiotic resistance. We found 16 resistance genes from *E. coli*, *S. aureus*, and *M. tuberculosis*, indicating that HGT played a key role in the development of these *Pseudomonas* strains. HGT can provide genes necessary for survival more quickly than spontaneous mutations (Charpentier et al., 2012). Although antibiotic resistance genes are an important public health concern in clinical and veterinary environments, it is unclear whether resistance genes of pathogenic microorganisms are transmitted from environmental bacteria or elsewhere (Forsberg et al., 2012). Therefore, HGT between *Pseudomonas* isolates and other bacteria should be further investigated.

## Conclusion

Knowledge of the antibiotic resistance profiles of food-related bacteria, including *Pseudomonas* spp., is currently needed. In this study, we successfully characterized the antibiotic susceptibility profiles of *Pseudomonas* spp. isolates from raw milk and assessed their antibiotic resistance determinants using whole genome sequencing. Our results indicate that MDR *Pseudomonas* species were prevalent in raw milk in Shaanxi province, China. Moreover, whole genome sequencing revealed the presence of different resistance determinants in all of the isolates as well as acquired resistance genes from other bacteria, demonstrating that HGT occurred between these *Pseudomonas* isolates and other bacteria. Although this study was conducted in only one region, the emergence of MDR *Pseudomonas* species and HGT is an important public health issue. Therefore, our data support the need for further investigations into *Pseudomonas* species to prevent the spread of resistance to other pathogenic bacteria.

## Data Availability Statement

The datasets generated for this study can be found in the NCBI Sequence Read Archive database (accession numbers PRJNA523883, PRJNA523885, and PRJNA593738).

## Author Contributions

LM and HL contributed equally, designed and performed the research. TL, LD, and HH helped with antimicrobial susceptibility testing. SZ and YZ helped with the data analysis. NZ gave advices to the researchers. JW gave opinions on the research design.

## Conflict of Interest

The authors declare that the research was conducted in the absence of any commercial or financial relationships that could be construed as a potential conflict of interest.

## Footnotes

1 www.i-sanger.com
