# Supplementary material for: Antibiotic Resistance Patterns of Pseudomonas spp. Isolated From Raw Milk Revealed by Whole Genome Sequencing
Source: Front Microbiol. 2020 Jun 3;11:1005. doi: 10.3389/fmicb.2020.01005 (PMC7326020; doi:10.3389/fmicb.2020.01005)
Supplement: Supplementary file 3 [file Table_3.DOCX]

Supplementary Table S3: The mutation identities of genes involved in antibiotic resistance comparing with *Pseudomonas aeruginosa* PAO1.

| Mutated Gene | Antibiotic Affected | Isolate | Mutation Identities | | | | Note |
| --- | --- | --- | --- | --- | --- | --- | --- |
|  |  |  | Nucleotide | E Value | Amino acid | E Value |  |
| *rps*L | streptomycin | 141-6 | 88% | 7e-052 | 92% | 2e-033 |  |
|  |  | 151-5 | 88% | 7e-052 | 92% | 2e-033 |  |
| PBP1a | β-lactams | 62-2 | 84% | e-112 | 74% | 0.0 |  |
|  |  | 141-6 | 85% | e-106 | 73% | 0.0 |  |
|  |  | 151-5 | 85% | e-106 | 74% | 0.0 |  |
| PBP1b | β-lactams | 62-2 | 87% | e-100 | 78% | 0.0 |  |
| PBP2 | β-lactams | 62-2 | 81% | e-172 | 79% | 0.0 |  |
| *opr*D | Imipenem, Meropenem | 141-6 | 87% | 1e-004 | 41% | 2e-093 |  |
|  |  | 151-5 | 87% | 9e-005 | 46% | e-103 |  |
| *gyr*A | Fluoroquinolones | 103-1 | 85% | 0.0 | 79% | 0.0 |  |
|  |  | 141-6 | 85% | 0.0 | 79% | 0.0 |  |
|  |  | 151-5 | 85% | 0.0 | 79% | 0.0 |  |
| *gyr*B | Fluoroquinolones | 141-6 | 85% | 0.0 | 86% | 0.0 |  |
|  |  | 151-5 | 85% | 0.0 | 85% | 0.0 |  |
| *par*C | Fluoroquinolones | 141-6 | 87% | e-167 | 85% | 0.0 |  |
|  |  | 151-5 | 87% | e-167 | 85% | 0.0 |  |
| *par*E | Fluoroquinolones | 151-5 | 85% | 0.0 | 89% | 0.0 |  |
| *glp*T | Fosfomycin | 141-6 | 85% | e-146 | 85% | 0.0 |  |
|  |  | 151-5 | 85% | e-146 | 85% | 0.0 |  |
| *mur*A | Fosfomycin | 141-6 | 84% | e-150 | 86% | 0.0 |  |
|  |  | 151-5 | 84% | e-150 | 86% | 0.0 |  |
| *pho*P | Polymyxin | 62-2 | 84% | e-116 | 86% | e-109 |  |
|  |  | 68-1 | 83% | e-101 | 84% | e-108 |  |
|  |  | 75-1 | 84% | e-115 | 85% | e-109 |  |
|  |  | 103-1 | 85% | e-122 | 85% | e-109 |  |
|  |  | 106-3 | 83% | e-101 | 84% | e-108 |  |
|  |  | 120-1 | 85% | e-127 | 84% | e-109 |  |
|  |  | 141-6 | 84% | e-112 | 85% | e-109 |  |
|  |  | 149-2 | 81% | 1e-072 | 83% | e-106 |  |
|  |  | 151-5 | 84% | e-112 | 85% | e-109 |  |
|  |  | 152-2 | 81% | 4e-070 | 83% | e-106 |  |
|  |  | 161-1 | 83% | 4e-039 | 83% | e-106 |  |
| *pho*Q | Polymyxin | 62-2 | 86% | 5e-029 | 67% | e-168 |  |
|  |  | 68-1 | 82% | 1e-020 | 67% | e-170 |  |
|  |  | 75-1 | 82% | 1e-022 | 66% | e-164 |  |
|  |  | 103-1 | 83% | 5e-025 | 66% | e-164 |  |
|  |  | 106-3 | 82% | 7e-021 | 67% | e-170 |  |
|  |  | 120-1 | 93% | 5e-025 | 66% | e-166 |  |
|  |  | 141-6 | 82% | 1e-022 | 67% | e-117 |  |
|  |  | 149-2 | 83% | 2e-021 | 66% | e-165 |  |
|  |  | 151-5 | 82% | 3e-020 | 66% | e-164 |  |
|  |  | 152-2 | 90% | 9e-020 | 66% | e-165 |  |
|  |  | 161-1 | 94% | 6e-024 | 66% | e-165 |  |
| *cls* | Daptomycin | 141-6 | 80% | 4e-035 | 71% | 0.0 |  |
|  |  | 151-5 | 80% | 4e-035 | 71% | 0.0 |  |
| *pgs*A | Daptomycin | 141-6 | 84% | 2e-059 | 81% | 7e-077 |  |
|  |  | 151-5 | 84% | 2e-059 | 81% | 7e-077 |  |
| *rpo*C | Daptomycin | 141-6 | 89% | 0.0 | 92% | 0.0 |  |
|  |  | 151-5 | 88% | 0.0 | 92% | 0.0 |  |
| *flol*R | Florfenicol | 141-6 | 86% | 2e-026 | 75% | 2e-083 |  |
|  |  | 151-5 | 86% | 2e-026 | 75% | 2e-083 |  |
| *rpo*B | Rifampicin | 141-6 | 87% | 0.0 | 92% | 0.0 |  |
|  |  | 151-5 | 87% | 0.0 | 92% | 0.0 |  |
| *sox*R | Global Regulatory Gene | 141-6 | 91% | 1e-013 | 59% | 1e-046 |  |
|  |  | 151-5 | 91% | 1e-013 | 59% | 1e-046 |  |
| *acr*R | AcrAB-TolC Hyperproduction | 141-6 | 41% | 2e-011 | 41% | 2e-043 |  |
|  |  | 151-5 | 41% | 2e-011 | 41% | 2e-043 |  |
| *nfx*B | MexCD-OprJ  hyperproduction | 68-1 | 83% | 2e-020 | 77% | 3e-073 |  |
|  |  | 106-3 | 83% | 1e-029 | 77% | 1e-079 |  |
|  |  | 120-1 | 93% | 5e-017 | 61% | 1e-057 |  |
| *mex*S | MexEF-OprN  Hyperproduction | 62-2 | 91% | 4e-004 | 58% | e-117 |  |
|  |  | 68-1 | 88% | 2e-006 | 59% | e-117 |  |
|  |  | 75-1 | 86% | 3e-004 | 59% | e-119 |  |
|  |  | 103-1 | 86% | 3e-004 | 59% | e-119 |  |
|  |  | 106-3 | 88% | 1e-006 | 58% | e-115 |  |
|  |  | 120-1 | 81% | 5e-006 | 58% | e-117 |  |
|  |  | 141-6 | 86% | 3e-004 | 63% | 6e-071 |  |
|  |  | 149-2 | 96% | 2e-004 | 59% | e-119 |  |
|  |  | 151-5 | 86% | 3e-004 | 59% | e-119 |  |
|  |  | 152-2 | 96% | 2e-004 | 59% | e-119 |  |
|  |  | 161-1 | 86% | 2e-004 | 58% | e-116 |  |
| *mex*L | MexJK Hyperproduction | 141-6 | 90% | 5e-011 | 62% | 8e-064 |  |
|  |  | 151-5 | 90% | 4e-011 | 62% | 8e-064 |  |
| *liaR* (*nar*L) | Daptomycin | 141-6 | 86% | 5e-011 | 61% | 3e-062 | LiaR / LiaS constitutes a two-component regulatory system in *Enterococcus faecium*. According to amino acid sequence, the corresponding protein sequences found in *Pseudomonas aeruginosa* PAO1 were NarL (protein homology 41.78%) and NarX (protein homology 49.09%). |
|  |  | 151-5 | 84% | 2e-016 | 63% | 3e-068 |  |
| *lia*S (*nar*X) | Daptomycin | 141-6 | 89% | 2e-013 | 39% | e-103 |  |
|  |  | 151-5 | 89% | 1e-013 | 39% | e-103 |  |
| *kas*A | Isoniazid | 141-6 | 59% | e-027 | 49% | e-100 | KasA was 3-oxoacyl-ACP synthase II in *Mycobacterium tuberculosis* H37Rv. According to amino acid sequence, the corresponding protein sequences found in *Pseudomonas aeruginosa* PAO1 was FabF (protein homology 41.29%). |
|  |  | 151-5 | 59% | e-027 | 49% | e-100 |  |
